# Aggressive Papillary Thyroid Carcinoma with Bilateral Internal Jugular Vein Invasion Initially Misdiagnosed as Nodular Goiter: A Multimodal Imaging and Molecular Case Report

**DOI:** 10.2174/0115734056469413260701105508

**Published:** 2026-07-09

**Authors:** Huili Pan, Zhiyan Luo, Yurong Hong, Pintong Huang

**Affiliations:** 1 Department of Nursing, The Second Affiliated Hospital Zhejiang University School of Medicine, Hangzhou, China; 2 Department of Ultrasound Medicine, The Second Affiliated Hospital Zhejiang University School of Medicine, Hangzhou, China

**Keywords:** Papillary thyroid carcinoma, Contrast-enhanced ultrasound, Vascular invasion, Tracheal invasion, BRAF mutation, TERT promoter, Multimodal imaging

## Abstract

**Introduction/Background:**

Papillary Thyroid Carcinoma (PTC) usually has an indolent course, but diffuse aggressive variants can mimic benign nodular goiter and lead to delayed diagnosis. Bilateral Internal Jugular Vein (IJV) invasion with tracheal infiltration represents exceptionally advanced disease. This case highlights how multimodal imaging and molecular profiling refine diagnostic and prognostic evaluation in such an aggressive PTC with tracheal and vascular invasion.

**Case Presentation:**

A 65-year-old woman was initially diagnosed with benign thyroid nodules based on ultrasound and CT. Progressive dyspnea prompted re-evaluation, during which multimodal imaging including conventional ultrasound, Contrast-Enhanced Ultrasound (CEUS), and contrast-enhanced CT revealed bilateral IJV invasion and tracheal destruction. Surgical intervention and pathology confirmed PTC harboring BRAF V600E and TERT promoter mutations. Early postoperative recurrence was observed.

The patient underwent radical thyroidectomy with careful assessment of vascular and tracheal involvement guided by imaging findings. Multimodal imaging and molecular profiling informed surgical planning and postoperative management.

Postoperative recovery was initially uneventful, with short-term follow-up showing no further immediate complications. Early recurrence emphasizes the aggressive nature of this tumor, while molecular findings provided prognostic insight.

**Conclusion:**

Diffuse aggressive PTC can mimic benign nodular goiter, leading to diagnostic delays. “Second-look” multimodal imaging combined with molecular analysis is essential for accurate diagnosis, risk stratification, and management planning. This case underscores the clinical importance of vigilance for vascular and tracheal invasion in PTC, particularly in atypical presentations.

## 1 INTRODUCTION

Papillary Thyroid Carcinoma (PTC) generally presents as a well-circumscribed, slow-growing nodule with an indolent clinical course. However, certain histopathological variants demonstrate diffuse infiltration and aggressive progression, occasionally mimicking benign conditions such as nodular goiter. Such atypical presentations often lead to misinterpretation, delaying accurate diagnosis and timely surgical intervention.

Vascular invasion in PTC, particularly involving the Internal Jugular Vein (IJV), is rare but clinically significant. Fewer than 30 cases of PTC with tumor thrombosis in regional veins, including the IJV, Superior Vena Cava (SVC), and ascending aorta, have been reported to date [1]. Although isolated IJV invasion has been reported in several case series, bilateral involvement remains exceptionally uncommon, representing a high-risk scenario that directly influences surgical planning and prognosis [2-7]. Subtle tumor thrombus may be overlooked, especially when the tumor obliterates the normal fat plane, creating the appearance of vessel-mass conflation. Recognition of bilateral IJV invasion requires meticulous imaging evaluation and often coordinated surgical resection with vascular specialists.

Ultrasound remains the first-line imaging modality for thyroid assessment, but conventional techniques may fail to detect diffuse or infiltrative disease. Contrast-Enhanced Ultrasound (CEUS) provides superior delineation of microvascular patterns and extrathyroidal extension, enhancing diagnostic accuracy and guiding surgical decision-making [8]. Complementary cross-sectional imaging with Contrast-Enhanced Computed Tomography (CECT) is particularly valuable for assessing large tumors, deep cervical spaces, or complex anatomy, allowing precise evaluation of tumor relationships with critical structures such as the trachea, esophagus, carotid sheath, and major veins [9].

Molecular profiling, including assessment of BRAF V600E and TERT promoter mutations, has emerged as a key adjunct in evaluating tumor aggressiveness and prognostic stratification.

Multiple studies have demonstrated that the co-occurrence of BRAF V600E and TERT promoter mutations predicts more aggressive disease, higher recurrence rates, and worse response to radioiodine therapy [10-17]. Recent guidelines also emphasize the integration of molecular data into risk stratification and individualized management plans for PTC [18].

Here, we report a rare and aggressive case of PTC initially misdiagnosed as nodular goiter, presenting with bilateral IJV invasion and tracheal infiltration without lymph node metastasis. Multimodal imaging combined with molecular analysis corrected the initial diagnostic oversight and guided tailored surgical management, highlighting important lessons for clinical practice regarding early recognition, surgical planning, and prognostic evaluation.

## 2 CASE PRESENTATION

This case report was approved by the Ethics Committee of the Second Affiliated Hospital of Zhejiang University School of Medicine, China, and the requirement for informed consent was waived (20251664). All procedures involving human participants were conducted in accordance with the ethical standards of the institutional ethics committee and with the Declaration of Helsinki.

### 2.1 Clinical History and Presenting Symptoms

A 65-year-old woman presented with a three-month history of progressive bilateral neck swelling. Initially, a local hospital diagnosed nodular goiter via ultrasound and CT, with no further intervention. The initial report did not identify overtly malignant features, and no biopsy was performed before referral. Two months later, she developed worsening dyspnea and palpitations. Repeat imaging revealed diffuse thyroid enlargement, prompting referral to our institution.

### 2.2 Past Medical History

Her past medical history included type 2 diabetes mellitus and cholecystectomy performed 22 years prior. She denied previous neck irradiation or family history of thyroid malignancy.

### 2.3 Clinical Findings on Examination

Bilateral, firm, and poorly mobile thyroid enlargement was palpable without evident cervical lymphadenopathy.

### 2.4 Diagnostic Assessment

#### 2.4.1 Laboratory Investigations

Serum thyroglobulin level was markedly elevated (161.3μg/L). Thyroid hormone and autoantibody levels were within reference ranges.

#### 2.4.2 Conventional Ultrasound

Ultrasonography revealed that the thyroid gland was enlarged with asymmetric bilateral lobes. The thyroid capsule appeared irregular, and the parenchyma demonstrated heterogeneous echogenicity with multinodular change. Focal protrusion of thyroid tissue into the bilateral internal jugular veins was noted, with posterior extension abutting the trachea, accompanied internally by coarse, strong echoes. Color Doppler imaging shows mixed intranodular vascularity. Cervical lymph nodes: Multiple oval hypoechoic lymph nodes were identified bilaterally. The largest nodes measure 0.89 × 0.40 cm (left level II) and 1.19 × 0.46 cm (right level II), with an additional node measuring 0.52 × 0.35 cm in right level IV. The nodes were circumscribed with homogeneous internal echotexture, and color Doppler demonstrates central (hilar) vascularity (Fig. **1A**-**H**).

#### 2.4.3 Contrast-Enhanced Ultrasound (CEUS)

CEUS showed a tumor thrombus in the left internal jugular vein exhibiting heterogeneous and hyperenhancement in the arterial phase, with intensity higher than that of the ipsilateral thyroid mass. In the venous phase, the tumor thrombus showed persistent enhancement with clear visualization of vascular invasion and helped differentiate tumor thrombus from bland thrombus because the lesion demonstrated internal perfusion rather than complete nonenhancement (Fig. **2A** and **B**). Because lymphoma was suspected and the mass was large, fine-needle aspiration was not performed; instead, a core-needle biopsy of the right neck mass was directly carried out, which confirmed papillary thyroid carcinoma.

### 2.5 Computed Tomography

Bilateral thyroid lobes were enlarged with heterogeneous attenuation. On contrast-enhanced images, the lesions demonstrated heterogeneous enhancement. The tracheal wall appeared irregular, with a focal intraluminal nodular protrusion. The lesion extended laterally to both sides of the neck and invaded the bilateral internal jugular veins, more pronounced on the right, where an intraluminal tumor thrombus was identified. The lesion showed close abutment to the strap muscles (Fig. **3A** and **B**). These findings suggested invasive disease rather than simple goiter, given the combination of diffuse enlargement, loss of normal tissue planes, venous invasion, and tracheal wall disruption.

### 2.6 Therapeutic Intervention

#### 2.6.1 Surgical and Pathological Findings

The patient underwent total thyroidectomy with bilateral lymph node dissection, partial resection of the internal jugular vein walls, and segmental tracheal resection with reconstruction. Intraoperatively, the tumor exhibited diffuse infiltration and firm adherence to strap muscles and the trachea. An intraluminal tumor thrombus was observed at the right internal jugular–superior vena cava junction and was successfully removed. The decision for combined resection was based on gross bilateral venous invasion and suspected airway infiltration on multimodal imaging, which made complete en bloc resection the most appropriate surgical strategy.

Histopathological evaluation confirmed papillary thyroid carcinoma with the typical papillary architecture and nuclear inclusions and was staged as T4aN0M0 according to the AJCC 8th edition, representing locally advanced disease with gross extrathyroidal extension. Lymphovascular invasion was histologically confirmed, while no regional lymph node metastasis was identified. Immunohistochemistry showed diffuse positivity for thyroglobulin, TTF-1 (SPT24), and CK-PAN. The Ki-67 proliferation index was 5%. Molecular testing identified BRAF V600E and TERT C250T promoter mutations (Fig. **4**).

### 2.7 Follow-up and Outcomes

The patient recovered uneventfully, but a contrast-enhanced CT scan at postoperative day 20 showed irregular soft-tissue density in the surgical bed with tracheal invasion and obliteration of vascular margins, suggestive of early recurrence. This early recurrence underscored the aggressive biological behavior associated with venous invasion and high-risk molecular alterations. Radioiodine-131 therapy was administered as postoperative adjuvant treatment; however, because this was a single case and complete longitudinal records were limited, detailed dose and cycle data were not available in the original dataset. After receiving iodine-131 therapy, she returned for a thyroid surgery follow-up one year later and was in good condition. Longer-term outcomes beyond one year were not available at the time of manuscript preparation, which is a limitation of this report.

## 3 DISCUSSION

Aggressive Papillary Thyroid Carcinoma (PTC) may rarely present as diffuse thyroid enlargement and mimic nodular goiter, particularly in the absence of a discrete dominant nodule. In such circumstances, grayscale ultrasound and color Doppler findings can appear deceptively benign, contributing to delayed recognition. In the present case, initial ultrasound and CT at an outside institution favored nodular goiter, whereas repeat (“second-look”) sonography at our center demonstrated focal protrusion into the Internal Jugular Vein (IJV) and clarified that the peri-airway echogenic foci reflected tracheal invasion rather than calcifications, highlighting the value of targeted re-evaluation when imaging and clinical evolution are discordant.

Compared with classic nodular goiter, aggressive PTC more frequently shows irregular capsule interruption, heterogeneous echogenicity, focal echogenic foci, abnormal internal vascularity, and tissue continuity with adjacent structures.

Because diffuse thyroid enlargement with vascular invasion and tracheal involvement is not specific to PTC, a broader differential diagnosis should also be considered. Follicular thyroid carcinoma with vascular invasion is an important alternative, as it shows greater propensity for hematogenous spread than classic PTC, though it lacks PTC’s characteristic nuclear features on pathology [19,20]. Thyroid lymphoma may present as a rapidly enlarging thyroid mass with compressive symptoms but typically demonstrates marked hypoechogenicity, diffuse infiltrative growth, and lymphoid histology. Anaplastic thyroid carcinoma should be considered in elderly patients with aggressive local invasion and airway compromise, though it usually shows marked cytologic pleomorphism, necrosis, and fulminant progression. Metastatic disease to the thyroid, particularly from renal cell carcinoma or gastrointestinal primaries, is another consideration; however, the absence of a known primary tumor, classic PTC morphology, and BRAF V600E/TERT promoter mutations support a primary thyroid origin in this case.

Although uncommon in differentiated thyroid cancer, venous tumor thrombus represents clinically significant vascular invasion that may limit the likelihood of complete resection and worsen prognosis. Proposed mechanisms include intravascular spread via the middle thyroid vein draining into the IJV, and reported risk factors include adult patients, advanced primary tumors (T3–T4), follicular carcinoma, aggressive PTC variants, Hürthle cell carcinoma, poorly differentiated or anaplastic carcinoma, and thyroid metastases from renal or colorectal primaries [21].

Multimodal imaging was essential for defining the extent of invasion and guiding operative strategy. Contrast-Enhanced CT (CECT) complemented ultrasound by delineating the anatomic extent of disease, confirming IJV invasion with intraluminal tumor thrombus, and further characterizing tracheal involvement. In addition, CECT can map caudal/mediastinal extension, and in extensive thrombi a “positive rim” sign may suggest preserved venous wall integrity and the technical feasibility of thrombectomy with or without vein sacrifice [20]. CEUS was particularly useful because it demonstrated enhancement within the thrombus, supporting tumor thrombus rather than bland thrombus and improving confidence in the diagnosis of vascular invasion.

Molecular profiling provided further prognostic stratification. The 2025 ATA guidelines (Recommendation 28) note that coexisting TERT promoter and BRAF V600E mutations are associated with worse prognosis, whereas isolated BRAF V600E is not consistently linked to aggressive behavior or inferior outcomes [18]. In this patient, the concurrent BRAF V600E and TERT promoter mutation was concordant with locally invasive disease and early postoperative recurrence, supporting risk-adapted surveillance and multidisciplinary management. Nevertheless, only two mutation sites were tested, so the full molecular landscape of the tumor could not be comprehensively characterized.

Overall, the present case is best framed as surgically locally advanced PTC, defined by gross extrathyroidal extension requiring combined resection and/or reconstruction (*e.g*., airway resection/reconstruction and major venous procedures such as thrombectomy, partial venous wall excision/repair, or vein sacrifice). Management in this setting prioritizes complete gross resection when feasible-typically total thyroidectomy with compartment-oriented neck dissection and en bloc resection of invaded structures followed by individualized adjuvant therapy and intensified follow-up [22,23], particularly in patients with high-risk molecular profiles and aerodigestive tract or major vascular invasion. Because this is a single-case report, the findings should be interpreted as hypothesis-generating rather than definitive evidence for treatment recommendations.

## CONCLUSION

Diffuse aggressive papillary thyroid carcinoma can closely resemble nodular goiter, concealing its malignant potential. Re-examining ultrasound data through a “second-look” approach and combining imaging with molecular analysis are essential for accurate diagnosis, prognosis, and management of these challenging cases. A multidisciplinary, image-guided, and mutation-aware approach is necessary for optimizing outcomes in these rare but aggressive cases. Long-term prognostic conclusions remain limited by the short follow-up period in this report.

## Figures and Tables

**Fig. (1):**
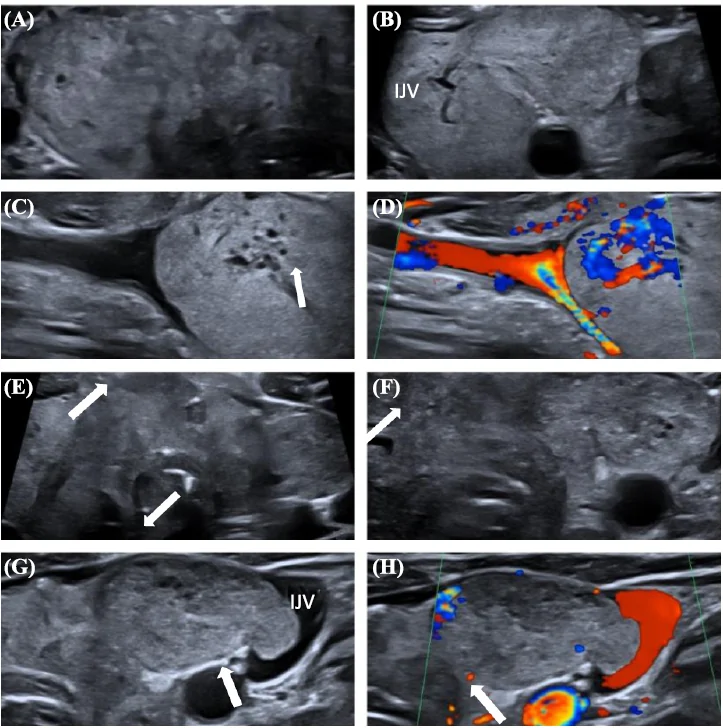
**A**. Transverse thyroid ultrasound: a diffuse mass in the right lobe of the thyroid. **B**. The mass in the right thyroid lobe extends to the ipsilateral internal jugular vein. **C**. Longitudinal scanning shows a tumor thrombus within the internal jugular vein. **D**. CDFI reveals a tumor thrombus occupying the right internal jugular vein, with residual blood flow signals detected within the remaining lumen. **E**. The thyroid isthmus shows a focal lesion with heterogeneous echogenicity; the posterior strong echoes indicate destruction of tracheal cartilage. **F**. Diffuse mass in the left thyroid lobe. **G**. Transverse thyroid ultrasound: the left-lobe mass extends to the ipsilateral internal jugular vein. **H**. CDFI demonstrates tumor thrombus in the left internal jugular vein with residual lumen blood flow signals.

**Fig. (2):**
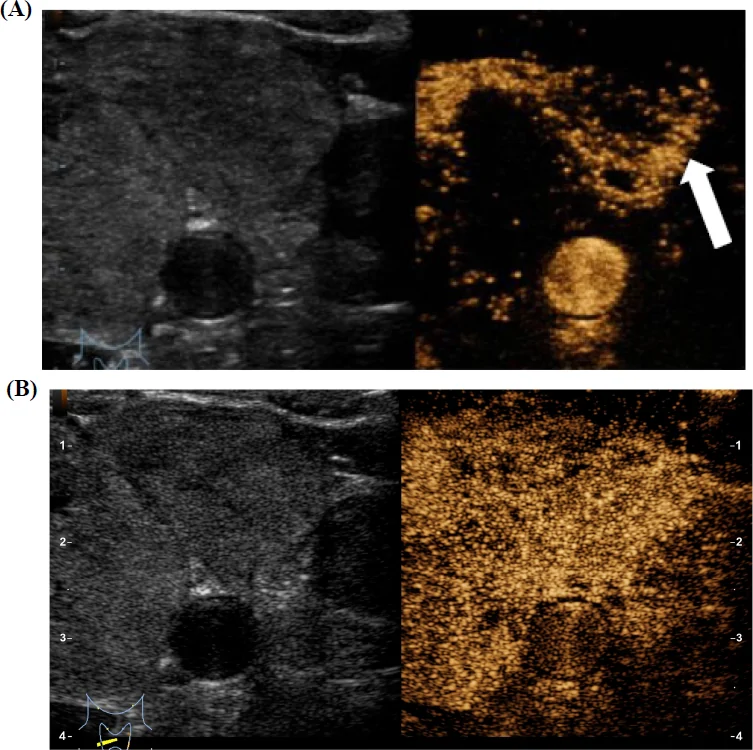
**A**: CEUS shows the tumor thrombus in the left internal jugular vein exhibiting hyperenhancement in the arterial phase, with intensity higher than that of the ipsilateral thyroid mass. **B**: The venous phase showed persistent enhancement with clear visualization of vascular invasion, particularly at the tumor-vessel interface.

**Fig. (3):**
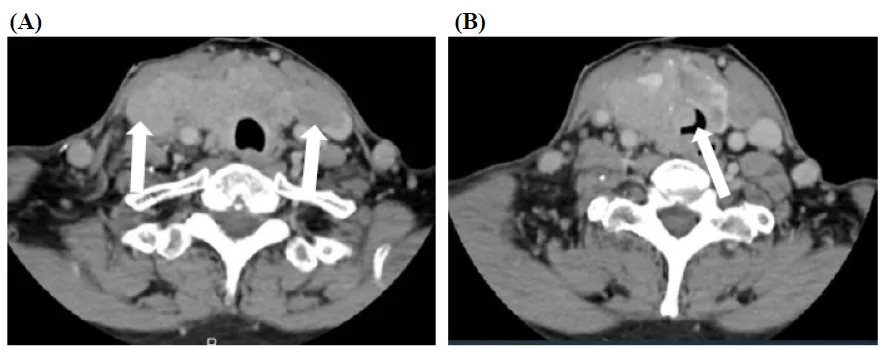
**A**: Contrast-enhanced neck CT shows diffuse thyroid enlargement with bilateral internal jugular vein invasion. **B**: The tracheal wall appears irregular, with a focal intraluminal nodular protrusion.

**Fig. (4):**
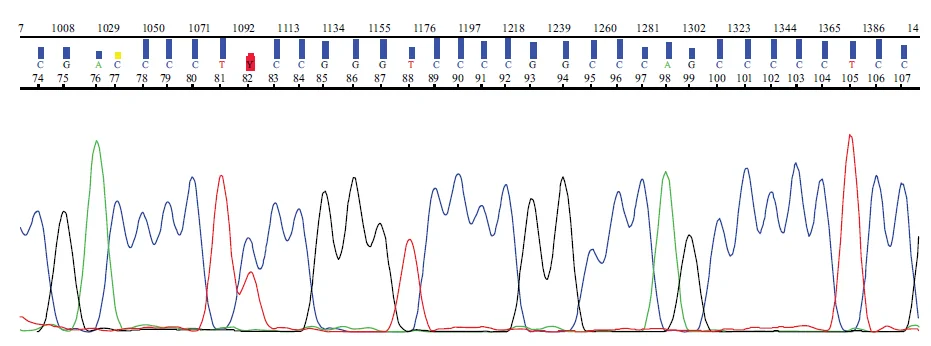
The sequencing chromatogram shows a positive TERT C250T mutation in this patient.

## Data Availability

All data supporting the findings of this study are contained within the article and its figures. De-identified imaging datasets (B-mode ultrasound, CEUS clips, and contrast-enhanced CT images), operative reports, and pathology/molecular results are available from the corresponding author [Y.H.] upon reasonable request. No proprietary code or custom software was used in this study.

## LIST OF ABBREVIATIONS

PTC: Papillary thyroid carcinoma
IJV: Internal jugular vein
CEUS: Contrast-enhanced ultrasound
CT: Computed tomography

## STANDARDS OF REPORTING

CARE guidelines were followed.
